# CROSSTALK BETWEEN UBIQUITINATION AND ADP-RIBOSYLATION IN THE MAINTENANCE OF MITOCHONDRIAL HOMEOSTASIS

**DOI:** 10.1093/geroni/igac059.1144

**Published:** 2022-12-20

**Authors:** Valentina Perissi

**Affiliations:** Boston University School of Medicine, Boston, Massachusetts, United States

## Abstract

Oxidative stress and increased ROS production, as observed in aging and obesity, commonly lead to the accumulation of mitochondrial dysfunctions. This is met by the activation of a robust mitochondria-to-nucleus stress response promoting the rewiring of nuclear gene expression to limit cellular and tissue damage and promote organelle adaptation. Here we will review previous work uncovering the transcriptional cofactor G-Protein Pathway Suppressor 2 (GPS2) as a mediator of mitochondria retrograde signaling and a key nuclear regulator of nuclear-encoded mitochondrial genes, including mitochondrial chaperones/proteases, mitokines, and other protective enzymes such as the ADP-ribosyltransferase NEURL4. We will also discuss unpublished work showing that shuttling of GPS2 between organelles plays a role in coordinating the transcriptional and translational regulation of antioxidant factors and pro-apoptotic genes by promoting the ubiquitination of mitochondria-associated translation factors.

